# Effects of Manufactured Sand and Steam-Curing Temperature on the Compressive Strength of Recycled Concrete with Different Water/Binder Ratios

**DOI:** 10.3390/ma16247635

**Published:** 2023-12-14

**Authors:** Xiaolin Liu, Xinjie Wang, Tianrui Zhang, Pinghua Zhu, Hui Liu

**Affiliations:** 1 School of Urban Construction, Changzhou University, 21 Gehu Middle Road, Wujin District, Changzhou 213164, China; s21030814004@smail.cczu.edu.cn (X.L.); s21030814002@smail.cczu.edu.cn (T.Z.); zph@cczu.edu.cn (P.Z.); liuhui@cczu.edu.cn (H.L.); 2 State Key Laboratory of Silicate Materials for Architectures, Wuhan University of Technology, Wuhan 430070, China

**Keywords:** water/binder ratio, steam-curing temperature, maturity–compressive strength relationship model

## Abstract

New building materials (manufactured sand and recycled coarse aggregates) can conserve raw materials and protect the environment. Prefabricated members can shorten the construction time of a structure. To use manufactured sand and recycled coarse aggregate in the preparation of precast member concrete, an economical and practical steam-curing scheme must be developed such that the compressive strength of precast manufactured sand recycled concrete (MRC) meets the requirements for hoisting. The effects of different steam-curing temperatures (standard curing, 40 °C, 50 °C, 60 °C, 70 °C, and 80 °C) on the compressive strength of MRC with three water/binder ratios (W/B) (0.46, 0.42, and 0.38) were studied. In addition, the microstructure of MRC was examined using a scanning electron microscope. The equivalent age–compressive strength model was used to estimate the recycled concrete with manufactured sand. The results showed that the strength of MRC with a water–cement ratio of 0.46, 0.42, and 0.38 reached 33.9, 38.7, and 45.1 MPa, respectively, after 28 days of standard curing. The results also indicated that an increase in the steam-curing temperature had a positive effect on the early compressive strength of MRC and a negative effect on the 28 d compressive strength. This behavior was more obvious for MRC with a low W/B ratio. For MRC with a W/B of 0.46, 0.42, and 0.38, after steam-curing for 6 h, the compressive strength reached 32–65%, 36–70%, and 40–77% of the design strength, respectively. The optimum steam-curing temperatures for MRC with W/B of 0.46, 0.42, and 0.38 were 60 °C, 60 °C, and 50 °C. A decrease in W/B has a negative impact on the accuracy of MRC estimation using the equivalent age–compressive strength model. The maximum deviation of the prediction was within 10%, and the accuracy of the model was acceptable. This study provides a useful reference for the production of prefabricated MRC components in factories and subsequent construction.

## 1. Introduction

The development of the construction industry has led to a shortage of natural materials, such as river sand (RS) and natural stones. In addition, many buildings must be dismantled, and an increasing amount of construction waste concrete is produced, resulting in environmental pollution. To solve these problems, manufactured sand (MS) and recycled coarse aggregates (RCA) have been proposed as replacements for river sand and natural coarse aggregates [1]. MS is a type of fine aggregate manufactured by crushing rock deposits with sharp edges and corners and a high fine powder content. RCA is produced by crushing construction waste into concrete. For the sustainable development of the construction industry, it is important to study the performance of recycled aggregate concrete (RAC) containing MS.

Researchers have recently suggested that the attached mortar in RCA is the principal reason for the reduction in the compressive strength of RAC [2,3,4]. The presence of attached mortar adversely affects the performance of RCA in terms of the crushing index, water absorption, and density [5]. These differences from natural coarse aggregates negatively affect the strength characteristics of RAC [6,7,8,9,10,11]. To improve the strength of recycled concrete, scholars usually use other types of cementing materials to replace cement. Lei et al. [12] found that adding fly ash with a 10% cement weight can significantly improve the strength and durability of recycled concrete. Mohmmad et al. [13] used geopolymer instead of cement to prepare geopolymer concrete with different proportions of nanosilica and found that GC can improve the rheological and mechanical properties of concrete. Recent studies have shown that adding appropriate amounts of kaolin clinker to recycled concrete can significantly increase its compressive and tensile strength, improve acid resistance, and increase overall durability [14]. The compressive strength of manufactured sand concrete (MSC) is controversial and may depend on the MS quality. Owing to the rough surface of MS, there is a high mechanical bite force between the MS particles, which improves the strength of the machine-made sand concrete under the same mix ratio [1,15]. Yang et al. [16] concluded that the incorporation of MS enhanced the strength of concrete, which was more evident with MS replacement greater than 40%. However, for MS with a high stone powder content, the addition of MS can reduce the compressive strength of MSC [17,18]. Feng et al. [18] found that when coarse aggregate is replaced by 100% RCA, the compressive strength of MSC is related to the quality and content of MS. With an increase in the replacement rate of medium-quality and low-quality MS, the strength first increased and then decreased; the strength increased with an increase in high-quality MS. It is necessary to further explore the change in the compressive strength of RAC mixed with MS.

With the mass production of prefabricated components, improving production efficiency is required. A common approach to enhancing the formwork turnover efficiency of prefabricated components and shortening the construction period is steam-curing. This method is often used to shorten precast concrete members to achieve demolding strength and lifting strength. At the end of steam-curing, the concrete strength is greatly enhanced, reaching more than 50% of the design strength [19,20]. The improvement in the early strength of concrete by steam-curing is because high temperatures accelerate the generation of hydration products [21,22] and reduce the later strength of concrete [23,24,25]. After curing at a constant temperature for a short time (6 h), the concrete should be maintained in a humid environment; otherwise, its long-term strength is reduced to a certain extent [24,26]. The steam-curing regime of concrete should be optimized to minimize the adverse impact of steam-curing. Based on natural concrete, the conclusion is that the pre-curing time should not be too short and the heating rate should be 10–20 °C/h [25,27]. Some conclusions have been drawn regarding MS and RCA concrete. Corominas et al. [28] revealed that the long-term strength of concrete prepared using low-quality RCA was slightly affected by steam-curing. Duan [29] et al. studied the effect of steam-curing on compressive strength based on MSC; it was concluded that when the steam-curing temperature was 40 °C, 50 °C, and 60 °C, it should be maintained for 6.2–31 h, 4–19 h, and 2.7–13 h, respectively. However, few studies have considered the effects of steam-curing on the compressive strength of concrete containing both MS and RCA.

Current research on the hydration products and compressive strength of steam-cured concrete is mostly based on natural concrete; a few studies were based on MSC or RAC. Research on manufactured sand and recycled aggregate concrete (MRC) has not been conducted in depth. Thus, this study considered the steam-curing of MRC and RAC with three water/binder ratios (W/B) and the effects of different steam-curing temperatures (standard curing, 40 °C, 50 °C, 60 °C, 70 °C, and 80 °C) and MS on the compressive strength of RAC. By observing the microstructure of 1 d concrete and the measured compressive strength of concrete at different ages (1, 3, 7, 14, and 28 d), the influence law of steam-curing on the MRC compressive strength with three W/B ratios and the optimal steam-curing temperature were obtained. To explore the effect of equivalent age on the strength of concrete at different steam-curing temperatures, the accuracy of the original equivalent age–compressive strength relationship model was verified.

## 2. Test

### 2.1. Test Materials

The properties of the concrete cementitious materials were tested. The apparent density of ordinary Portland cement 42.5 (P.O42.5) from the Jiangsu Jinfeng Cement Group was 3050 kg/m^3^, and the apparent density of Class I dry-discharge fly ash (FA) produced by the Changzhou Hutang Thermal Power Plant was 2500 kg/m^3^. Other properties of the cementitious materials are presented in Table 1. 

**Figure 1:**
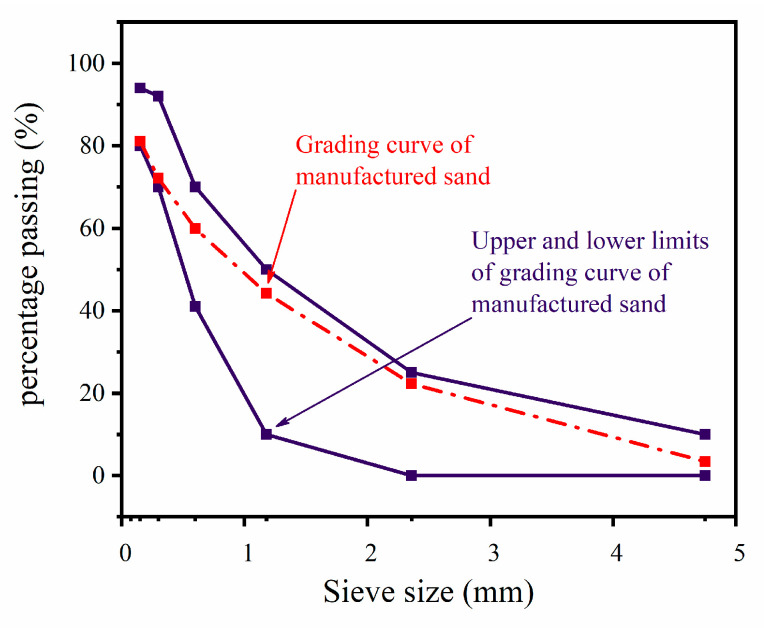
Sieve size: grain gradation of MS.

The particle gradation curves of the MS are shown in Figure 1. The aggregates were tested; the details are presented in Table 2. MS was provided by Changzhou Anfeng Technology Co., Ltd. (Changzhou, China). Except for the methylene blue value (MB), the MS properties met the requirements of the specification [30]. The RCA was provided by Jiangsu Wujin Green and was produced and provided by Lvhe Environmental Technology Co., Ltd. (Changzhou, China); its performance met the requirements [31].

### 2.2. Mix Ratio

The concrete mix ratio was calculated according to the total amount calculation method [32]. According to this method, when the W/B ratios are 0.46, 0.42, and 0.38, the design strengths of concrete are C30, C35, and C40, respectively. The additional water consumption of the RAC and MRC was calculated according to the additional water consumption method. The mix ratios of concrete with different W/B ratios are presented in Table 3 (RAC30 and MRC30 represent W/B of 0.46; RAC35 and MRC35 represent W/B of 0.42; and RAC40 and MRC40 represent W/B of 0.38). 

### 2.3. Experimental Method

#### 2.3.1. Concrete Mixing Method

Concrete mixing uses a two-stage mixing method. MS and RCA were poured into the HJW60 concrete test mixer and mixed for 30 s until well-mixed. Additional water was added and stirred for 60 s until all the water was absorbed by the MS and RCA. The fly ash and cement were poured into the concrete mixer, half of the water was added, and the mixture was stirred for 60 s until there was no dry powder. The remaining water and water reducing agent were added to the mixture and stirred for 90 s. After stirring evenly, the mixture was placed in a mold, placed on a vibrating table, and vibrated for 20 s to remove air bubbles and improve the compactness of the concrete. The concrete was smoothed after the initial coagulation (after 1 h). At this stage, the temperature was maintained at 20 ± 2 °C.

#### 2.3.2. Steam-Curing Conditions

**Figure 2:**
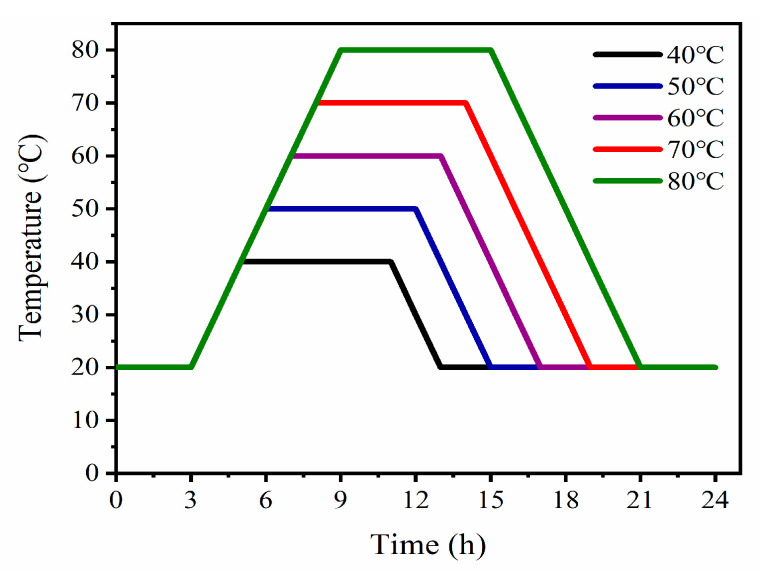
Steam-curing system.

Of the six concretes, one group of concrete test blocks (three test blocks) was put in a standard curing room (20 ± 2 °C) for standard curing (numbered as 0). The other five groups of concrete were put into a ZKY-400 steam-curing box for steam-curing (40 °C, 50 °C, 60 °C, 70 °C, and 80 °C, numbered 1–5, respectively). The concrete surface was covered with a PVC film to reduce thermal damage from steam-curing. Steam-curing includes four stages: pre-curing, temperature increase, constant temperature, and temperature decrease. After the initial setting, the concrete was immediately placed in a steam-curing box, and the temperature was maintained at 20 °C for 3 h. Subsequently, the temperature was increased to 40 °C, 50 °C, 60 °C, 70 °C, or 80 °C at a heating rate of 10 °C/h [25,27]. The final temperature of the steam-curing box was maintained for 6 h. Ultimately, the temperature was reduced to 20 °C at a cooling rate of 10 °C/h. The concrete block was removed from the steam-curing box and demolded at room temperature. After demolding, the test blocks were immediately sent to the standard curing room. The steam-curing regime for the experiment is shown in Figure 2.

#### 2.3.3. Mechanical Strength Test

At 1, 3, 7, 14, and 28 d of standard curing and steam-curing, compressive strength tests of the six types of concrete were conducted. The concrete test blocks were crushed using a WA-600C electro-hydraulic servo universal testing machine. The loading rate was 0.5 MPa/s. The compressive strength test blocks were 100 mm × 100 mm × 100 mm, and the test was carried out following GB/T 50081-2019 “Standard for Test Methods for Physical and Mechanical Properties of Concrete” [33]. 

#### 2.3.4. SEM

The microstructure of the samples was photographed by a scanning electron microscope (SEM, SUPRA55, Zeiss, Jena, Germany). Each concrete sample with a size of approximately 0.5 cm × 0.5 cm was knocked out of the 1 d compressive strength test blocks with a hammer. The hydration reaction was stopped by immersing the sample in absolute ethanol for 7 d. The samples were placed in an oven at 40 °C for dry treatment. Before testing, the samples were sprayed with gold to enhance their electrical conductivity. The samples were observed under an electron microscope at 1000×.

## 3. Results and Discussion

### 3.1. The 28 d Compressive Strength of Concrete with Different W/B

**Figure 3:**
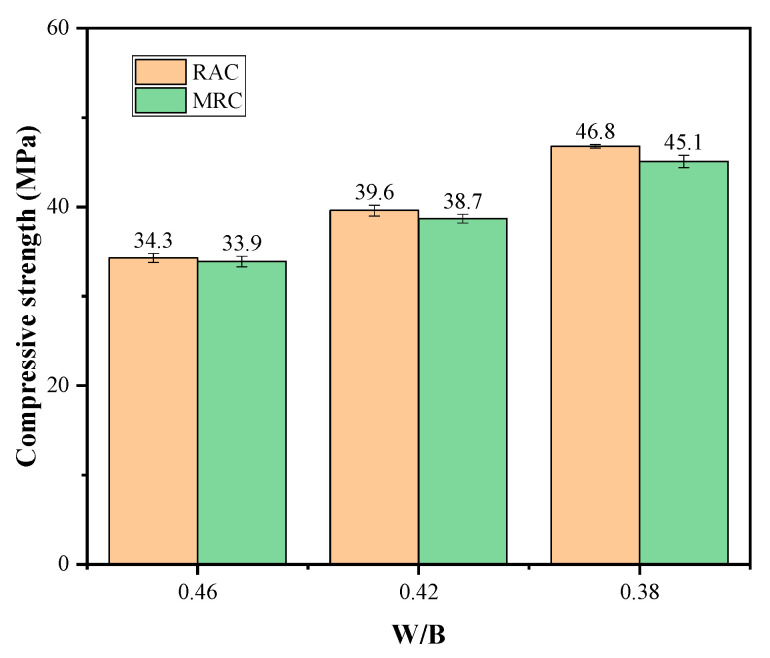
The 28 d compressive strength of MRC and RAC with standard curing.

The 28 d compressive strengths of RAC and MRC with different W/B ratios in standard curing conditions are shown in Figure 3. The 28 d compressive strength of the RAC reached 110% of the design strength. Although the MS quality was poor, the 28 d compressive strength of the MRC reached the design value. With the same W/B, the compressive strength of MRC was slightly lower than that of RAC (the difference in strength between the two was not more than 5%). Most studies have shown that the rough surface and polygonal shape of MS are beneficial for improving the connection between the cementitious material and aggregate, increasing the compressive strength [15,16]. A few studies have shown that the compressive strength of MRC first increases and then decreases with an increase in low-mass MS [18]; however, it is not clear which specific index of the physical properties of MS causes the decrease in MRC compressive strength. Comparing the performance of the manufactured sand in this experiment with past experiments, it was found that the physical properties of the manufactured sand in this experiment, in addition to the MB value (the size of the MB value reflects the content of mud powder in the manufactured sand), and other MS properties were improved. However, the compressive strength of concrete still decreased, suggesting that a larger MB value may negatively impact the compressive strength of MRC containing 100% MS. The MB value of MS was 1.7, which indicates a relatively large proportion of mud powder in the fine powder of MS and a relatively small proportion of stone powder. Absorbed water results in a weakening of the cement bonding ability. This is the fundamental reason why the MRC strength was lower than that of the RAC. It was also found that the 28 d compressive strength of the MRC decreased more rapidly with a lower W/B. With the W/B ratio from 0.46 to 0.38, the compressive strength of the MRC decreased by 0.4 MPa, 0.9 MPa, and 1.7 MPa, respectively.

### 3.2. Development Law of Compressive Strength with Different Curing Temperatures

**Figure 4:**
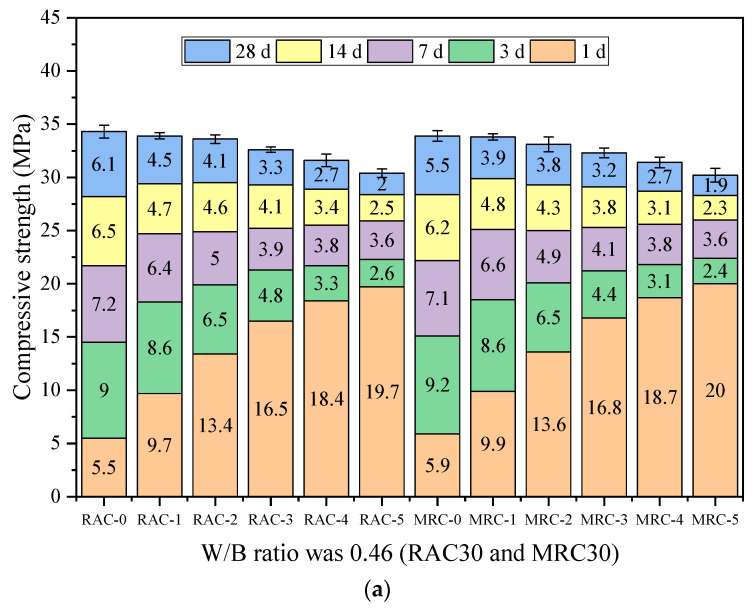

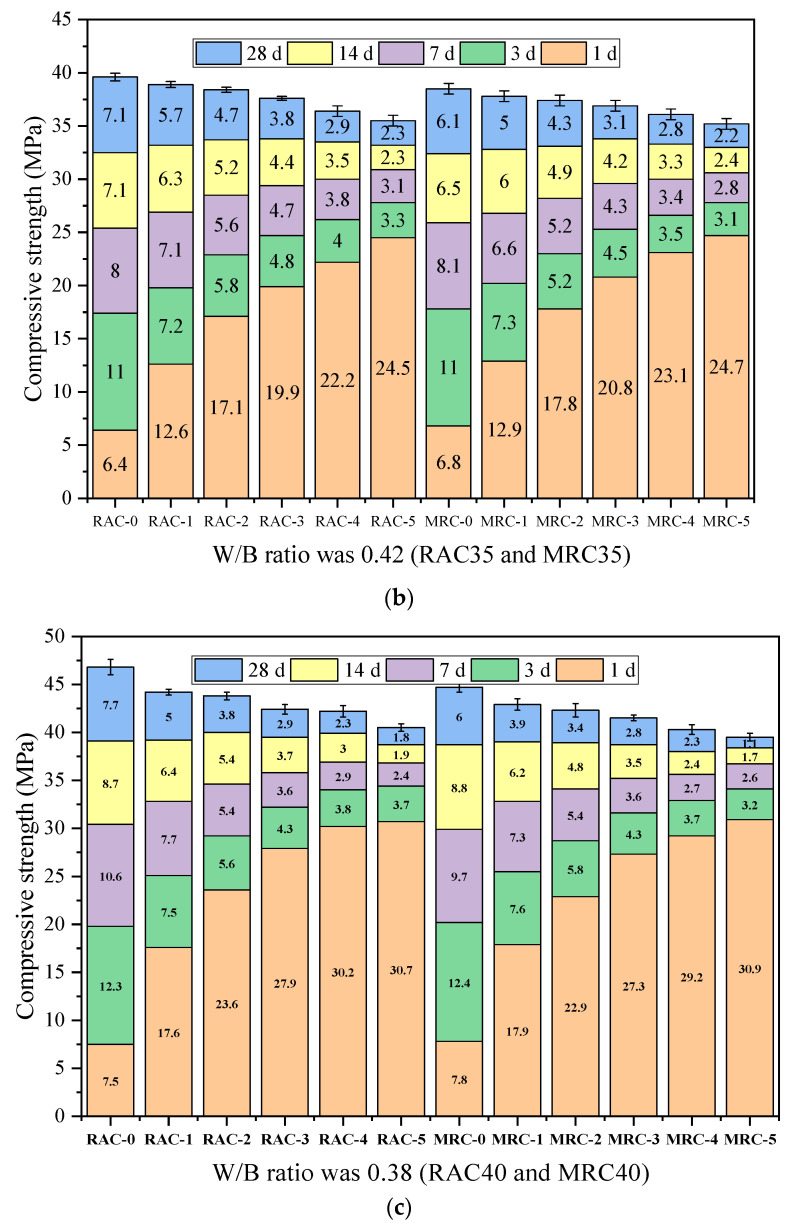
Compressive strength of concrete of different ages at different temperatures: (**a**) W/B ratio was 0.46; (**b**) W/B ratio was 0.42; (**c**) W/B ratio was 0.38.

Figure 4 shows the development law of the compressive strength of concrete with three W/B ratios at different steam-curing temperatures. In the early stage, for both MRC and RAC, the compressive strength was significantly higher with steam-curing than with standard curing. At 1 d, the compressive strengths of MRC30 and MRC35 were slightly higher than those of RAC under the same conditions. However, at 28 d, the compressive strength of the MRC was slightly lower than that of the RAC under the same conditions. 

Taking MRC30 as an example, from 40 °C to 80 °C, the 1 d compressive strength gradually increased. The 1 d compressive strength obtained under steam-curing is 1.68, 2.31, 2.85, 3.17, and 3.39 times that under standard curing, and 66.67% of the design strength, respectively. At 60 °C and above, the 1 d compressive strength of MRC30 was sufficient to meet the demolding strength requirements of prefabricated components (prefabricated components require demolding strength to reach 50% of the design strength). At all steam-curing temperatures, the 7 d compressive strength of the MRC reached 75% of the design strength (prefabricated components require hoisting strength to reach 75% of the design strength). At this time, the concrete with standard curing did not reach 75% of the design strength; thus, it could not be hoisted. The compressive strength growth slowed significantly after 7 d. With standard curing, the 28 d compressive strength of MRC30 reached 113% of the design strength. With increasing curing temperature, the 28 d compressive strength of MRC30 gradually decreased. The compressive strength of the concrete at each steam-curing temperature reached 111.67%, 110.33%, 107.67%, 104.67%, and 100.67% of the design strength, respectively.

It is observed that excessive temperature is not conducive to the 28 d compressive strength of MRC. The effects of steam-curing on the development laws of the compressive strengths of MRC and RAC are similar. Similar trends were observed, except that the early intensity of MRC40 was roughly the same as that of RAC under the same conditions. The higher the steam-curing temperature, the more unfavorable the effect on the later strength. The results with MS and RCA used in concrete were similar to research results from previous studies based on natural concrete [23,24,25]. With a rapid increase in the gel structure, the inner CSH gel generates a large amount of gel water on the inner and outer surfaces of the cement stone [25]. The high temperature causes the gel water to be heated and evaporated into water vapor, destroying the internal structure of the cement stone, which significantly reduces the growth rate of the concrete strength in the later stage.

### 3.3. Influence of Temperature on the Compressive Strength of Concrete with Different W/B

**Figure 5:**
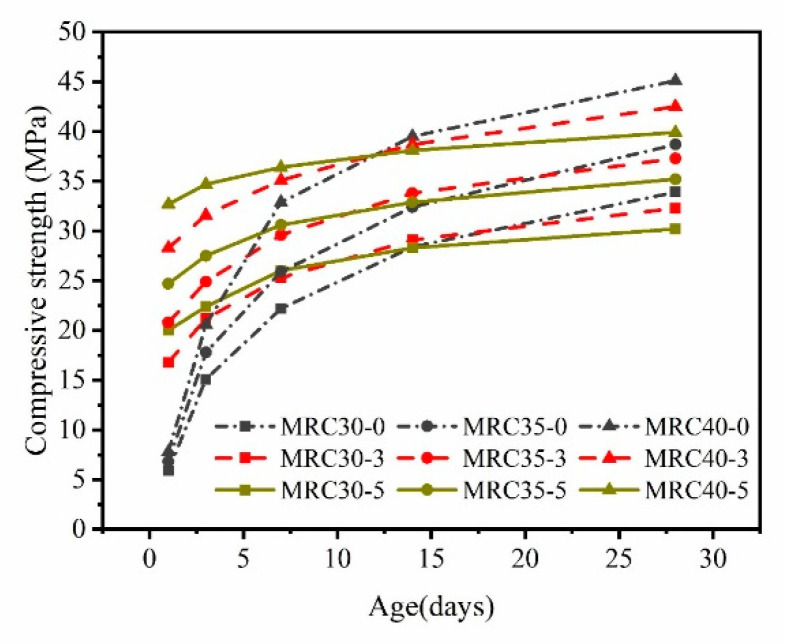
Changes in compressive strength with aging of concrete with different W/B.

**Figure 6:**
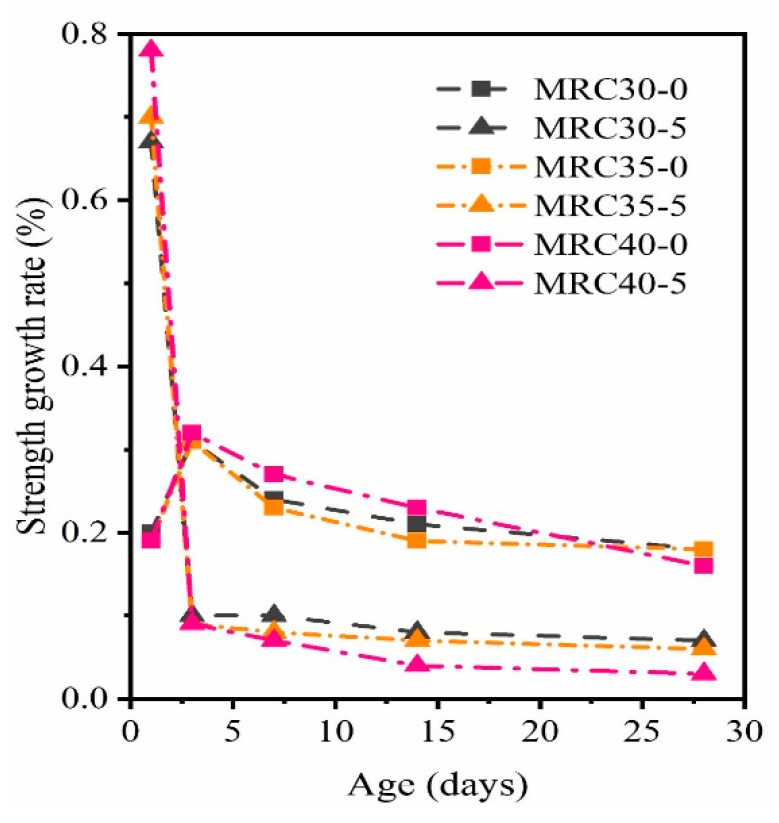
Growth rate of compressive strength of MRC with different W/B.

Figure 5 and Figure 6 show the effect of the steam-curing temperature (three typical curing temperatures) on the compressive strength of concrete with different W/B ratios and the growth rate of the compressive strength at different ages. In Figure 5, at 80 °C, the 28 d compressive strength of MRC40 did not reach 40 MPa (39.7 MPa), and the 28 d compressive strength of MRC30 and MRC35 barely reached the design strength. Compared with standard curing, the compressive strength decreased. As the W/B ratio decreased, the compressive strength increased.

Figure 6 shows that with standard curing, the growth rates of the three W/B concrete at each age were essentially the same. However, with steam-curing at 80 °C, the growth rate of early strength with different W/B (0.38, 0.42, and 0.46) decreased with an increase in the W/B ratio. The 1 d compressive strength reached 40–77%, 36–70%, and 32–65% of the design strength, respectively. With an increase in age, the smaller the W/B ratio, the smaller the later growth rate of the MRC. At the highest temperature of 80 °C, the later strength growth of concrete with a W/B of 0.38 was close to 0. Increased temperature in steam-curing has a significant negative effect on the compressive strength of MRC in the later stage with a low W/B ratio. In actual production, to reduce the adverse effect on the later strength, the steam-curing temperature should be as low as possible to shorten the construction period. Thus, different steam-curing temperatures should be used for MRC with different W/B ratios. MRC with W/B ratios of 0.46, 0.42, and 0.38 should be steam-cured at 60 °C, 60 °C, and 50 °C, respectively.

### 3.4. Microstructure of Early MRC

**Figure 7:**
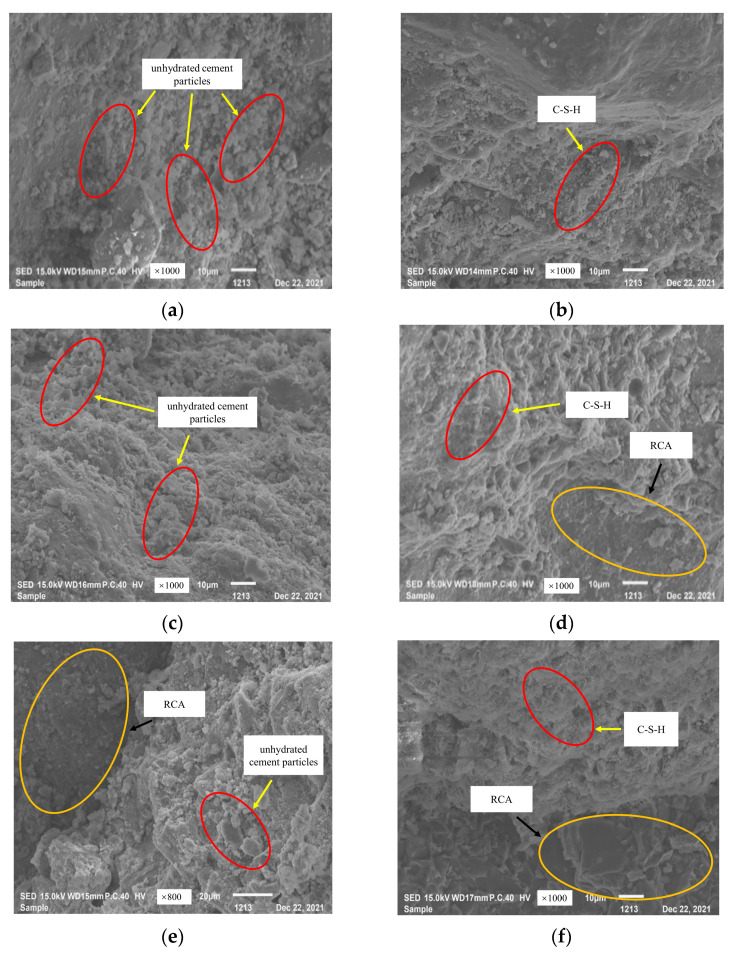
Early hydration products of MRC at different temperatures: (**a**) MRC30 Standard curing; (**b**) MRC30 60 °C; (**c**) MRC35 Standard curing; (**d**) MRC35 60 °C; (**e**) MRC40 Standard curing; (**f**) MRC40 60 °C.

Figure 7 shows the 1 d hydration products of three W/B of MRC at standard curing temperature and 60 °C steam-curing temperature. In Figure 7a,c,e with standard curing, the unhydrated cement has more particles, a more dispersed floc structure distribution, and a smaller volume of floc structure. With steam-curing, in Figure 7b,d,f, MRC30 and MRC35 still have some unhydrated cement particles after 1 d of steam-curing at 60 °C but are not hydrated. The cement particle content decreased significantly, and the volume of the flocculent structure increased. This indicates that the improvement in early strength of low W/B ratio concrete (MRC30 and MRC35) with steam-curing at 60 °C is mainly due to the increase in the volume of hydration products. Figure 7f shows the hydration product of MRC40 with 60 °C steam-curing for 1 d. Figure 7a shows that MRC40 still has unhydrated cement particles after 1 d of steam-curing at 60 °C, but compared with the concrete with standard curing, the hydration reaction rate is increased owing to the increase in curing temperature. The hydration products are a large number of floc structure crystals (CSH) in the concrete; they are intertwined and are an important support for the strength of the concrete. This indicates that the improvement in the early strength of MRC40 concrete with 60 °C steam-curing is due to the increased volume of hydration products and to the change in the morphology of the hydration products.

### 3.5. Equivalent Age–Compressive Strength Model of MRC

As it is difficult to test the strength of concrete before demolding, it is important to determine when to demold and lift to avoid failure to meet the strength requirements. The classic maturity theory can be used to predict the strength of concrete. Some researchers established the relationship between concrete strength and maturity by defining the total temperature over a certain period as maturity [34]. Others subsequently proposed an equivalent-age calculation method based on the Arrhenius function. The concrete age at any curing temperature is converted to the age under the reference temperature difference, known as the equivalent age. Steam-curing of concrete is a complicated humidity- and temperature-coupling environment [24,35]. In addition to curing temperature and time, humidity also affects the strength of concrete [34,36]. Most maturity–strength calculation models are based on water curing or a 100% relative humidity environment [37]. However, the curing of many concrete components in actual projects involves curing in the natural environment at a relatively low humidity; thus, the existing maturity model overestimates the late strength of the concrete.

Equation (1) [38] was used to convert the age at each curing temperature to the equivalent age at the reference temperature of 293 K (20 °C); the temperature is the thermodynamic temperature. The concrete strength at each curing temperature was calculated using the following formula:

(1) $$t_{e}=\sum_{0}^{t}\exp[-\frac{E_{a}}{R}(\frac{1}{T+273}-\frac{1}{T_{r}+273})]\Delta t=\sum f(t)\Delta t,$$

where: 

*t_e_*—equivalent age (d or h);

*E_a_*—the ‘‘apparent” activation energy (J/mol);

*R*—the universal gas constant (J/K·mol);

*T_r_*—the average temperature of concrete during a time interval (K);

*T*—the specified temperature (K);

Δ*t*—the time interval (d or h).

RILEM [39] Technical Committees recommend *E_a_/R* to be 4000 for Portland cement (P·I or P·II) and slag cement (P·S) (when the temperature is greater than or equal to 20 °C). The calculated equivalent age of the concrete at each stage is presented in Table 4.

Researchers have proposed different strength–maturity empirical relationship models based on their research and analysis. This study used a function model in ASTM-C1074 for MRC fitting [40]:

(2) $$S=S_{u}\frac{k(t_{e}-t_{0})}{1+k(t_{e}-t_{0})}$$

where:

*S* is the compressive strength at age *t_e_* (MPa);

*S_u_* is the limiting strength (MPa);

*k* is the rate constant (1/d);

*t_e_* is the test equivalent age (d);

*t*_0_ is the age at which strength development begins (d). 

**Figure 8:**
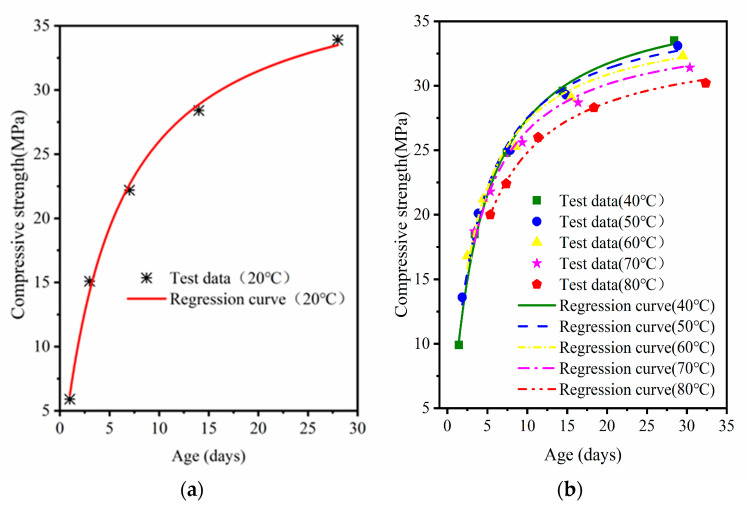
MRC30 equivalent age–compressive strength model verification: (**a**) standard curing; (**b**) 40 °C–80 °C.

**Figure 9:**
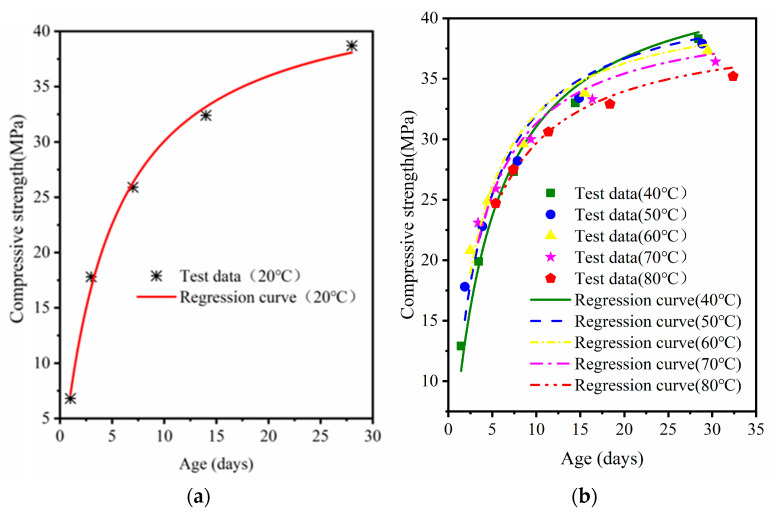
MRC35 equivalent age–compressive strength model verification: (**a**) standard curing; (**b**) 40 °C–80 °C.

**Figure 10:**
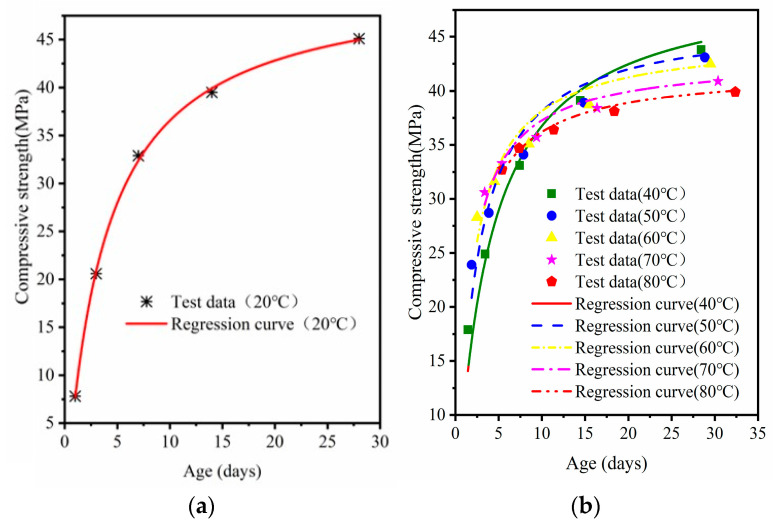
MRC40 equivalent age–compressive strength model verification: (**a**) standard curing; (**b**) 40 °C–80 °C.

Equation (2) was used to fit the compressive strength of MRC at standard curing, 40 °C, 50 °C, 60 °C, 70 °C, and 80 °C curing versus age. The fitted curves are shown in Figure 8, Figure 9 and Figure 10; the regression parameters are presented in Table 5.

Differences in the curing temperature and W/B ratio can lead to reduced ultimate strength of high temperature cured concrete [34]. In Table 4, the ultimate strength for each W/B ratio decreases with an increase in temperature. With a decrease in W/B, the amount of ultimate strength loss also increases; the ultimate strength with 0.38 W/B loses nearly 10 MPa. The correlations between the fitting curves of the three W/B concretes and the experimental data gradually decreased with increasing steam-curing temperatures. The *R^2^* of MRC30 was 0.98–0.99; the *R*^2^ of MRC35 was 0.96–0.99. The fitting results of MRC40 also had a good correlation but were slightly inferior to those of MRC30 and MRC35 (*R*^2^ was 0.93–0.98). The smaller the W/B, the more obvious the decrease in *R*^2^ with increasing temperature. 

**Figure 11:**
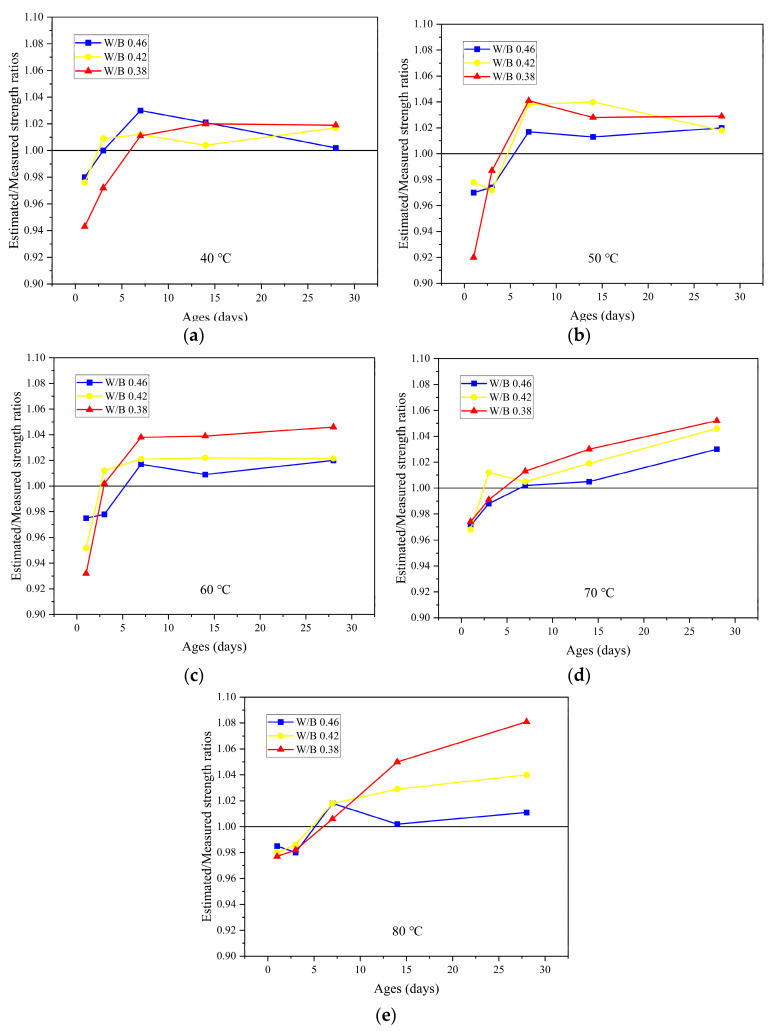
Model accuracy: (**a**) 40 °C; (**b**) 50 °C; (**c**) 60 °C; (**d**) 70 °C; (**e**) 80 °C.

Figure 11 depicts the accuracy of the predicted values with different W/B at the same curing temperature to determine the prediction accuracy of the model for the compressive strength at a specific age. The results show that the early strength of concrete estimated by the equivalent age–compressive strength relationship model is lower than the measured value, and the estimated later strength is higher than the measured value. As the predicted value of the early intensity of the model is lower than the measured intensity, it is conservative for practical application; thus, the model can be applied to the early intensity estimation of the MRC. For the later strength, the accuracy of the model is better at a curing temperature of 40–70 °C; at 80 °C, the predicted value of concrete with a W/B ratio of 0.38 is 8% higher than the measured value. Thus, the model is not suitable for estimating the late compressive strength of MRC with higher curing temperatures. For MRC with a W/B ratio of 0.46 and 0.42 at a curing temperature of 40–70 °C, the error of the predicted value of the model for each age is within 5%, indicating good applicability.

## 4. Conclusions

In this paper, the steam-curing of MRC with different W/B ratios was carried out based on recycled concrete with manufactured sand. The effect of steam-curing temperature on the early and late compressive strengths of MRC with different W/B ratios was studied, and the accuracy of the equivalent age–compressive strength relationship model for estimating the compressive strength of MRC was verified. The conclusions are presented as follows:

(1) The addition of MS increased the compressive strength of RAC at early ages and decreased the 28 d compressive strength of RAC, irrespective of the curing regime;
(2) The early compressive strength of the MRC increased rapidly with increasing steam-curing temperatures. More hydration products and needle-like ettringite were found with higher steam-curing temperatures at 1 d, which explains the higher early compressive strength of MRC with steam-curing;
(3) For the same steam-curing temperature, the hoisting strength (75% of the design compressive strength) can be obtained earlier with a smaller W/B ratio. The optimum steam-curing temperatures of MRC with W/B ratios of 0.46, 0.42, and 0.38 were 60 °C, 60 °C, and 50 °C, respectively;
(4) With an increase in the W/B ratio, the correlation of the fitting curve of the MRC equivalent age–compressive strength relationship model gradually worsened but still had a good correlation (*R*^2^ greater than 0.93). The model underestimates early strength and overestimates late strength. Except at 80 °C, the deviation between the measured and estimated values was within 5%, indicating good applicability of the model.

## Figures and Tables

**Table 1:** Chemical composition of Portland cement and fly ash (wt%).

	Loss on Ignition (%)	SO_3_ (%)	Al_2_O_3_ (%)	Fe_2_O_3_ (%)	SiO_2_ (%)	CaO (%)	Specific Surface Area (m^2^/kg)	3 d Flexural Strength (MPa)	3 d Compressive Strength (MPa)
Cement	4.02	1.81	7.33	3.75	21.35	60.71	360	5.3	26.5
Fly ash	1.8	0.8	28.31	3.69	52.32	3.81	470	/	/

**Table 2:** Properties of constituent aggregates of concrete.

Material	Apparent Density (kg/m^3^)	Fineness Modulus	Crushing Value (%)	Stone Powder Content (%)	MB ^a^	24 h Water Absorption (%)	Adsorption Mortar Content (%)
RS	2589	2.4	17.4	/	/	/	/
MS	2669	2.7	24.5	10.6	1.7	5.4	/
RCA	2520	/	16.2	/	/	2.6	32

^a^ MB: methylene blue value.

**Table 3:** Mix proportions (kg/m^3^).

Concrete	W/B	RCA	Fine Aggregate		Cement	FA	Water	Additional Water	Water Reducing Agent
			RS	MS					
RAC30	0.46	956.9	753.2	/	288.5	123.6	189.6	20.1	3.24
MRC30	0.46	956.9	/	760	288.5	123.6	189.6	46.7	3.24
RAC35	0.42	963.3	744.5	/	303.2	129.9	181.9	20.3	3.42
MRC35	0.42	963.3	/	753.7	303.2	129.9	181.9	46.1	3.42
RAC40	0.38	987.9	721.5	/	318	136.3	172.6	20.8	3.63
MRC40	0.38	987.9	/	728.9	318	136.3	172.6	44.5	3.63

RS: river sand, MS: manufactured sand, FA: fly ash.

**Table 4:** Concrete equivalent age table (days).

Time	Standard Curing	40 °C	50 °C	60 °C	70 °C	80 °C
Before heating	/	0.125	0.125	0.125	0.125	0.125
After heating	/	0.26	0.37	0.52	0.73	1.41
Before cooling	/	0.85	1.26	1.81	2.56	3.96
After cooling	/	0.99	1.50	2.21	3.17	5.25
1 d	1	1.44	1.87	2.50	3.38	5.37
3 d	3	3.44	3.87	4.50	5.38	7.37
7 d	7	7.44	7.87	8.50	9.38	11.37
14 d	14	14.44	14.87	15.50	16.38	18.37
28 d	28	28.44	28.87	29.50	30.38	32.37

**Table 5:** Regression parameters of strength versus time at different curing temperatures.

Temp (°C)		Regression Parameters			
		*S_u_* (MPa)	*k* (1/day)	*t*_0_ (day)	*R* ^2^
MRC30	standard	38.87856	0.18754	0.01611	0.99473
	40	37.52722	0.2703	0.07573	0.99687
	50	35.89052	0.3223	0.08424	0.99193
	60	34.56026	0.3334	0.09971	0.9862
	70	33.7804	0.3492	0.16252	0.98993
	80	33.40744	0.3530	0.49494	0.98893
MRC35	standard	44.65274	0.20758	0.07925	0.98298
	40	44.3649	0.22326	0.01412	0.98315
	50	42.91311	0.29345	0.02541	0.95013
	60	41.64182	0.33739	0.03412	0.96176
	70	40.72913	0.33432	0.04243	0.95594
	80	39.64537	0.29914	0.05004	0.96384
MRC40	standard	51.36229	0.25461	0.30459	0.98876
	40	50.29198	0.27147	0.02547	0.97887
	50	46.71335	0.44744	0.06578	0.95535
	60	44.93006	0.56294	0.04156	0.94568
	70	42.99299	0.65348	0.0425	0.92492
	80	41.54725	0.62614	0.02667	0.92975

## Data Availability

The data used to support the findings of this study are available from the corresponding author upon request.
